# Australian endemic pest tephritids: genetic, molecular and microbial tools for improved Sterile Insect Technique

**DOI:** 10.1186/1471-2156-15-S2-S9

**Published:** 2014-12-01

**Authors:** Kathryn A Raphael, Deborah CA Shearman, A Stuart Gilchrist, John A Sved, Jennifer L Morrow, William B Sherwin, Markus Riegler, Marianne Frommer

**Affiliations:** 1 Evolution and Ecology Research Centre, School of Biological, Earth and Environmental Sciences, The University of New South Wales, Sydney, NSW 2052, Australia; 2 Hawkesbury Institute for the Environment, University of Western Sydney, Locked Bag 1797, Penrith, NSW 2751, Australia

## Abstract

Among Australian endemic tephritid fruit flies, the sibling species *Bactrocera tryoni *and *Bactrocera neohumeralis *have been serious horticultural pests since the introduction of horticulture in the nineteenth century. More recently, *Bactrocera jarvisi *has also been declared a pest in northern Australia. After several decades of genetic research there is now a range of classical and molecular genetic tools that can be used to develop improved Sterile Insect Technique (SIT) strains for control of these pests. Four-way crossing strategies have the potential to overcome the problem of inbreeding in mass-reared strains of *B. tryoni*. The ability to produce hybrids between *B. tryoni *and the other two species in the laboratory has proved useful for the development of genetically marked strains. The identification of Y-chromosome markers in *B. jarvisi *means that male and female embryos can be distinguished in any strain that carries a *B. jarvisi *Y chromosome. This has enabled the study of homologues of the sex-determination genes during development of *B jarvisi *and *B. tryoni*, which is necessary for the generation of genetic-sexing strains. Germ-line transformation has been established and a draft genome sequence for *B. tryoni *released. Transcriptomes from various species, tissues and developmental stages, to aid in identification of manipulation targets for improving SIT, have been assembled and are in the pipeline. Broad analyses of the microbiome have revealed a metagenome that is highly variable within and across species and defined by the environment. More specific analyses detected *Wolbachia *at low prevalence in the tropics but absent in temperate regions, suggesting a possible role for this endosymbiont in future control strategies.

## Introduction

**Figure 1:**
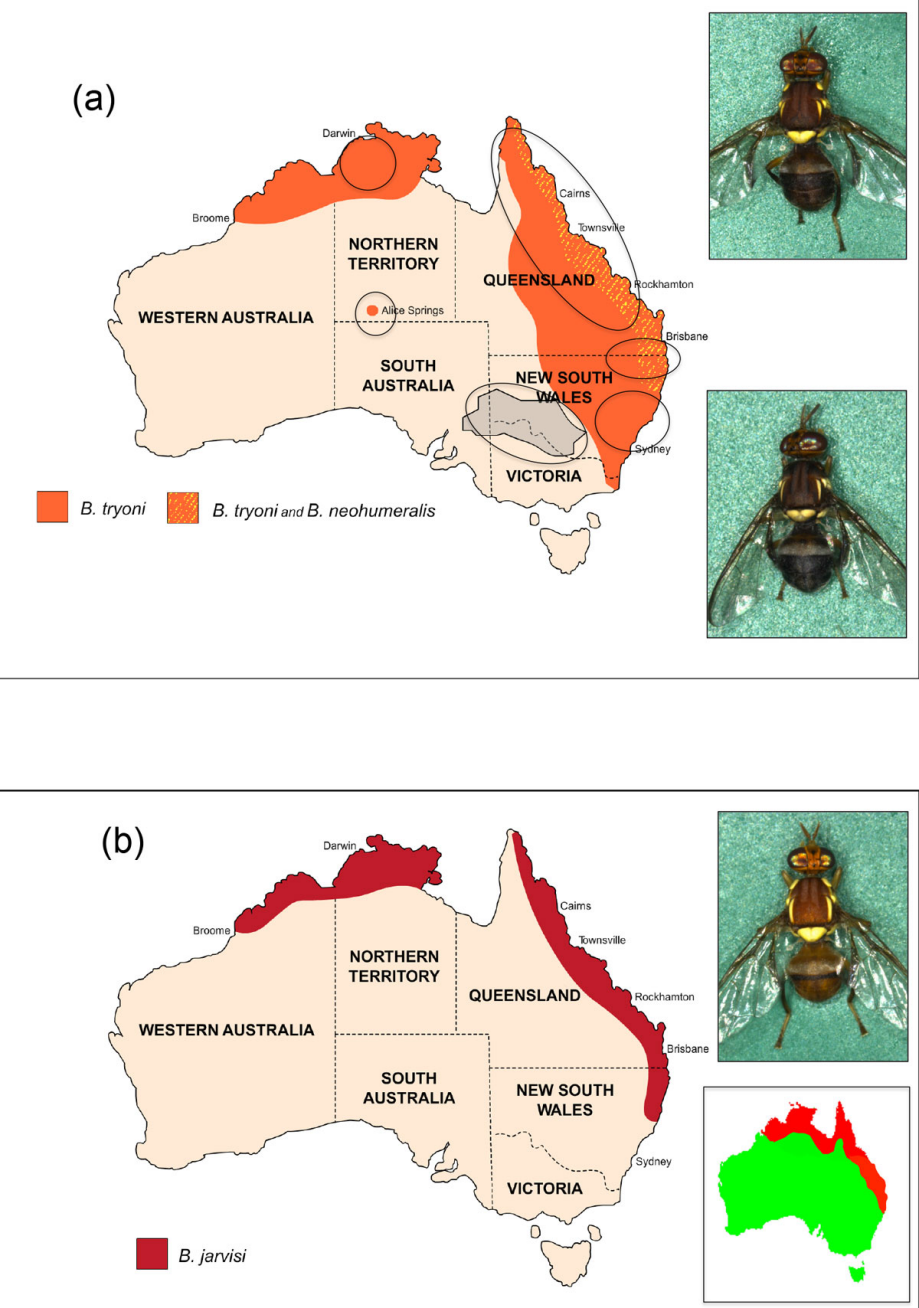
**The distributions of *B. tryoni, B. neohumeralis *and *B. jarvisi *in Australia**. (a) The distribution of *B. neohumeralis *is entirely within the broader distribution of *B. tryoni*. The ovals show the populations of *B. tryoni *that are differentiated by microsatellite analyses. Grey shading is the FFEZ (the Fruit Fly Exclusion Zone). Top right*, B. tryoni*, bottom right, *B. neohumeralis*. (b) The distribution of *B. jarvisi *and, inset, the distribution of its native host the Cocky apple (*Planchonia careya*). Top right, *B. jarvisi*.

The family Tephritidae includes some of the most significant pests of horticulture in the world. Among tephritid fruit flies endemic to Australia, the sibling species, *Bactrocera tryoni *(Queensland fruit fly, Qfly) and *Bactrocera neohumeralis *(lesser Queensland fruit fly), have both been serious pests since the establishment of horticulture in Australia in the nineteenth century. Both species are polyphagous, infesting a very broad range of cultivated fruits and vegetables [1]. However, *B. tryoni *is considered the more serious pest because, in contrast to *B. neohumeralis*, it is highly invasive and has followed the spread of horticulture through eastern Australia [2], including into drier and cooler areas beyond its native habitat (Figure 1a). More recently, *Bactrocera jarvisi *has been declared a pest in northern Australia [3], although this species has a narrower host range [1]. Its distribution largely follows that of its native host, the Cocky apple (*Planchonia careya*), but within this region it will infest cultivated mango and guava [4] (Figure 1b).

The traditional form of pest control has been the use of insecticidal cover sprays and baits, but there is now a worldwide move to ban many insecticides, so the need for more environmentally acceptable forms of control is clear. The Sterile Insect Technique (SIT) involves the area-wide release of huge numbers of sterile insects to overflood a wild pest population. The mating of sterile males with wild females results in the collapse of the wild population over successive releases [5]. SIT, as an area-wide form of insect control, can in favourable conditions eliminate pest insects from both horticultural areas and refugia. Additionally, in contrast to insecticides, SIT is species-specific and it is unlikely for insects to become resistant. Disadvantages include the expense and technical difficulties of establishing and maintaining a mass-rearing facility, the need to mark released flies to distinguish them from wild flies in the field, the reduced fitness of mass-reared flies in the field due to laboratory adaptation and the effects of radiation. Amongst *Bactrocera*, the successful eradication of *Bactrocera cucurbitae *from the Okinawa islands of Japan provides a benchmark for SIT programs with mixed sex releases [6].

So far, to control *B. tryoni*, SIT has been employed at a relatively small scale in Australia, in an area that covers temperate fruit growing regions of the states of New South Wales, Victoria and South Australia, formerly designated the Fruit Fly Exclusion Zone (FFEZ, Figure 1a), and also to control isolated outbreaks in South Australia and Western Australia. These regions have been favourable for SIT as they provide only marginal habitat for *B. tryoni *and, for this reason, outbreaks (as determined by regulatory authorities) are limited in spread and often involve very small numbers of flies [7].

A notable success story for SIT against Qfly is its eradication from Perth, Western Australia in 1995 [8]. Other small-scale field trials in inland regions of eastern Australia could not demonstrate clear reduction of population numbers [9], possibly because of the poor survival of released flies to mating age and natural reduction in the wild population due to the very dry climate of the treatment areas. SIT was used annually within the FFEZ from 1996, however, despite quarantine and control measures, outbreaks of Qfly now occur most years within the zone; from July 2013 Area Freedom was lost from the FFEZ, being retained only in the Greater Sunraysia Pest Free Area, a small FFEZ sub-area of the Murray Valley [10]. The entire state of South Australia is also free of Qfly and has Area Freedom but is vulnerable to outbreaks.

The reasons for loss of Area Freedom in the FFEZ are many, including the risk of migration of flies from surrounding areas, leading to outbreaks within the FFEZ, and the restrictions on the use of insecticides that reduce the ability to control flies in the areas that surround the FFEZ. Climate change may also be contributing to the ability of flies to establish in and adapt to previously marginal habitat [11]. As Dominiak and Ekman [12] point out, the options for fruit fly control are becoming ever more limited with the withdrawal of many pesticides by regulatory authorities, and there is a need for the integration of different control strategies such as Integrated Pest Management or IPM, including aspects of biological control [13,14]. SIT is one of the strategies that is easily incorporated into IPM. This review will focus on the use of genetics to improve SIT against endemic *Bactrocera *pest flies in Australia, including the use of traditional genetic crosses to improve SIT strains, the use of molecular genetic techniques to mark release strains and develop male-only strains, and the new disciplines of genomics, transcriptomics and microbiomics to increase our knowledge of these pest species and inform the development of new control methods.

## Improved mass rearing of SIT strains by hybridisation of domesticated strains

The standardised conditions in a mass-rearing facility are clearly quite different to the variable conditions that wild flies experience in the field, and this results in strong selection as field populations adapt to laboratory conditions. A number of life history traits have been observed to change as a result of domestication including earlier age of reproductive maturity, increased fecundity, more rapid development time, and reduced lifespan and stress resistance. These changes have been reported in *B. tryoni *[9,15] as well as other tephritids including medfly, *Ceratitis capitata *[16], olive fruit fly, *Bactrocera oleae *[17], and melon fly, *Bactrocera cucurbitae *[18].

In Qfly, Meats et al. [15] showed that increased productivity occurs rapidly, in the first four generations after domestication, and Gilchrist et al. [20,21] have repeatedly shown that factory strains are inbred. Indeed, Dominiak [19] observed the "self-defeating" nature of ever-increasing production levels that jeopardise fly quality in the Qfly mass rearing facility.

**Figure 2:**
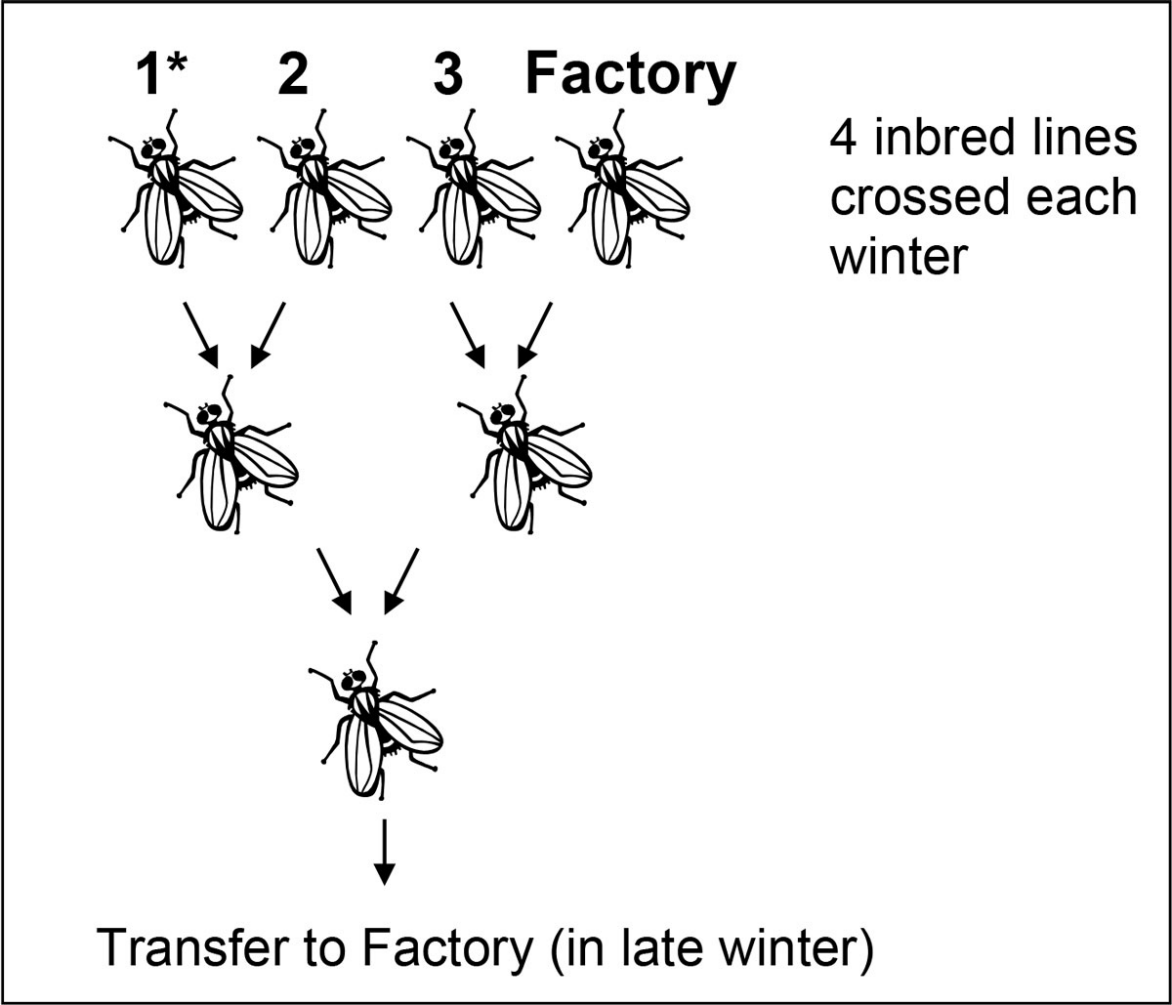
**The 4-way crossing scheme to produce an outbred Factory strain **[9]. Three domesticated (inbred) strains are maintained in the Factory and crossed following the illustrated scheme with the fourth strain coming from the mass-reared flies. Genetic markers can be incorporated via one of the inbred strains (1*). If the marker is mitochondrial DNA, then 1* must be all female and in the second generation cross the females must be the daughters of the marked females in generation 1.

Introducing wild flies into the mass-reared strains every year is ineffective since the mass-reared strain immediately out-competes the wild flies, and simply repeats the problem of inbreeding after domestication. A solution to this problem was proposed by Gilchrist [9] who used a 4-way crossing scheme designed to allow simple and rapid annual replacement of mass-reared strains in even small facilities. The scheme, which involves crossing 3 partially inbred lines with an existing mass-reared strain (Figure 2), avoids the problem of inbreeding and lab adaptation while maintaining high productivity. Because these flies were outbred, their field performance approached that of wild flies, showing improved longevity, temperature stress resistance and dispersal, all important post-release fitness characters that allow released males to compete with wild flies in the field. Significantly, the high productivity shown by these hybrid strains was equal to that of the existing mass-reared strain, meaning that even with annual replacement, the facility will suffer no loss of output [22]. The scheme could be compatible with the incorporation of a genetic-sexing strain (GSS).

## Genetically marked strains

Released flies are normally marked with fluorescent dust in order to distinguish them from wild flies when both are caught in monitoring traps. However a small proportion of sterile flies can escape being marked by this method or they lose the dust after release, and sometimes the dust can be transferred to a wild fly when both are trapped together. This becomes critical where SIT is used in an area that is being monitored for Area Freedom and misidentification of a sterile fly as a wild fly can lead to loss of market access. In addition, the fluorescent dust is considered a hazard for personnel working in the mass-rearing facility, so a more effective and less hazardous marking technique is being sought.

DNA microsatellites have been used to distinguish trapped flies of uncertain origin [20,23-26], but while microsatellites can be useful for identifying released sterile flies, markers that are unique to the released flies are preferable as markers for SIT strains. The ratio of the isotopes ^13^C:^12^C and ^15^N:^14^N clearly separates wild and factory flies in both *B. tryoni *and *C. capitata *[9,27]. However, this is an expensive technique to employ for day to day monitoring and relies on ready access to specialist personnel and equipment. Below we discuss two newer alternatives: genetic transformation and interspecific or interstrain crosses.

### (i) Transformation with molecular markers

**Figure 3:**
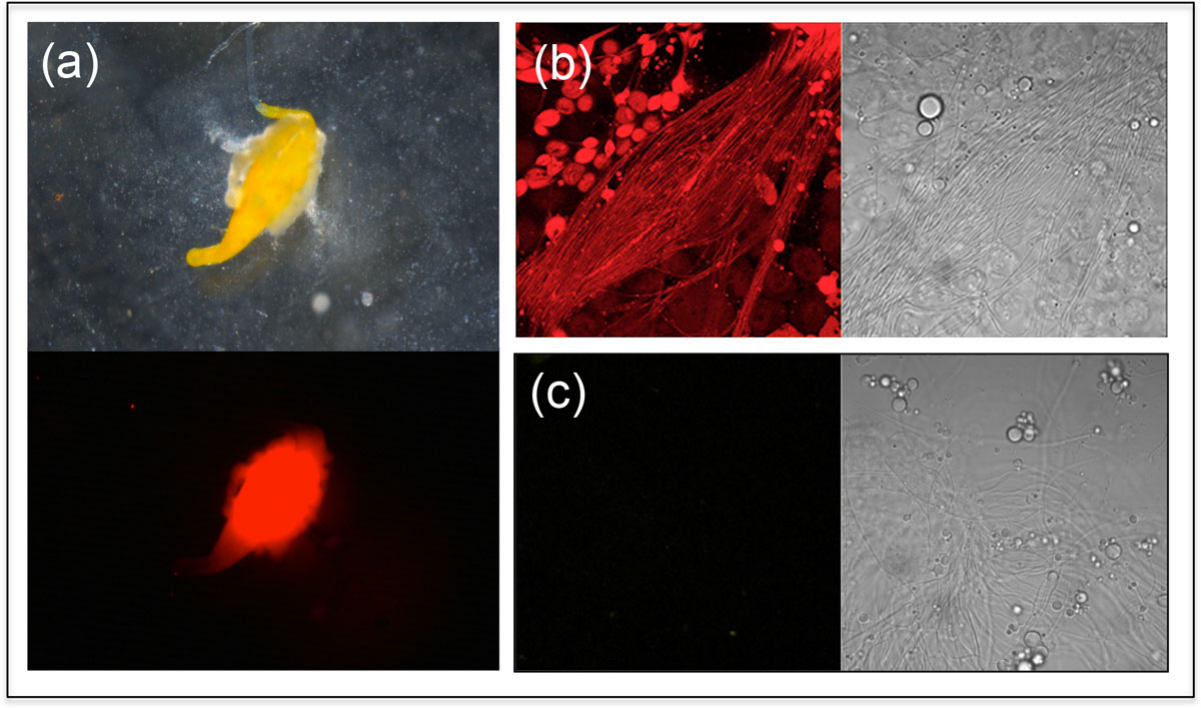
**Fluoresence in testes and sperm from *B. tryoni *transformed with vector 1261**. (a) A testis dissected from a 1261 transformed fly. Top, white light; bottom, with dsRED Ultra filter. (b) and (c) Confocal images of sperm from a 1261 transformed male (b) and a non-transformed male (c). Sperm were observed using an inverted Zeiss LSM 5 laser scanning confocal microscope. In each case left is the fluorescence image, the right image is bright field.

Germ-line transformation is now routine for many non-Drosophilid species and could be used to introduce heritable genetic markers into SIT strains as well as to develop male-only broods. Genetic transformation of *B. tryoni *has been established [28] using *piggyBac *as a vector. Flies were marked with the fluorescent proteins EGFP or DsRed under the control of the *Drosophila *polyubiquitin promoter [29,30], providing the possibility for integrated fluorescent markers. These same vectors have been modified to carry in addition the genes encoding either turboGFP or DsRedExpress markers linked to a *C. capitata *β*2-tubulin *spermatogenesis-specific promoter (the 1260 and 1261 vectors respectively), as well as an incorporated *attP *for site-specific integration, and *gypsy *insulator elements [31], and have been used to successfully mark sperm in *C. capitata *[31]. When microinjected into *B. tryoni *eggs, only one of the two vectors was successfully incorporated into the germ-line (Table 1). The flies had green fluorescence in the body and red fluorescence in dissected testes and sperm (Figure 3). The results demonstrated that the *C. capitata *β2-tubulin promoter works successfully in the genus *Bactrocera*. The fluorescent sperm marking make this a useful strain for sperm transfer studies.

**Table 1:** Gene transformation results.

Vector number	No. eggs injected	No. G0 adults emerged	% survival	No. G1 adults screened	No. G1 fluorescent flies
1260	896	77	8.6	2974	0
1261	823	55	6.7	2729	2*

Two vectors, 1260 (pBac{<af_attP-Ccb2t-tGFP_af<_PUb- DsRed}) and 1261 (pBac{>f_attP-Ccb2t-DsRedEx_a>_PUbnlsEGFP}) [31] were microinjected into *B. tryoni *eggs with phsp-pBac transposase as helper as in [28].

* from a single G0 male

### (ii) Markers from interspecific crosses

An alternative to germ-line transformation for the introduction of genetic markers is to use interspecific or interstrain crosses to incorporate a unique section of a donor DNA into the target SIT strain. Such markers may be useful where regulatory approval for genetically modified organisms is not available or difficult to obtain. Interspecific hybrids between *B. tryoni *and *B. jarvisi *have been used to introduce novel markers into *B. tryoni *[32]. *B. tryoni *and *B. jarvisi *have been classified into separate sub-genera of the *Bactrocera *genus [33]. The two species have distinct morphology and behaviours, including host fruit preference and lure attractancy [4]. DNA evidence [34] has confirmed that there are substantial differences between the species, but not sufficient to merit the different subgeneric status. Cruikshank *et al*. [35] showed, surprisingly, that the two species can be crossed in the laboratory to produce fertile hybrids. This crossing capacity has provided the ability to produce genetically-marked strains that could potentially be useful for sterile release, thereby avoiding the necessity for using genetically-transformed strains and allowing rapid incorporation of genetic markers into sterile release strains.

**Figure 4:**
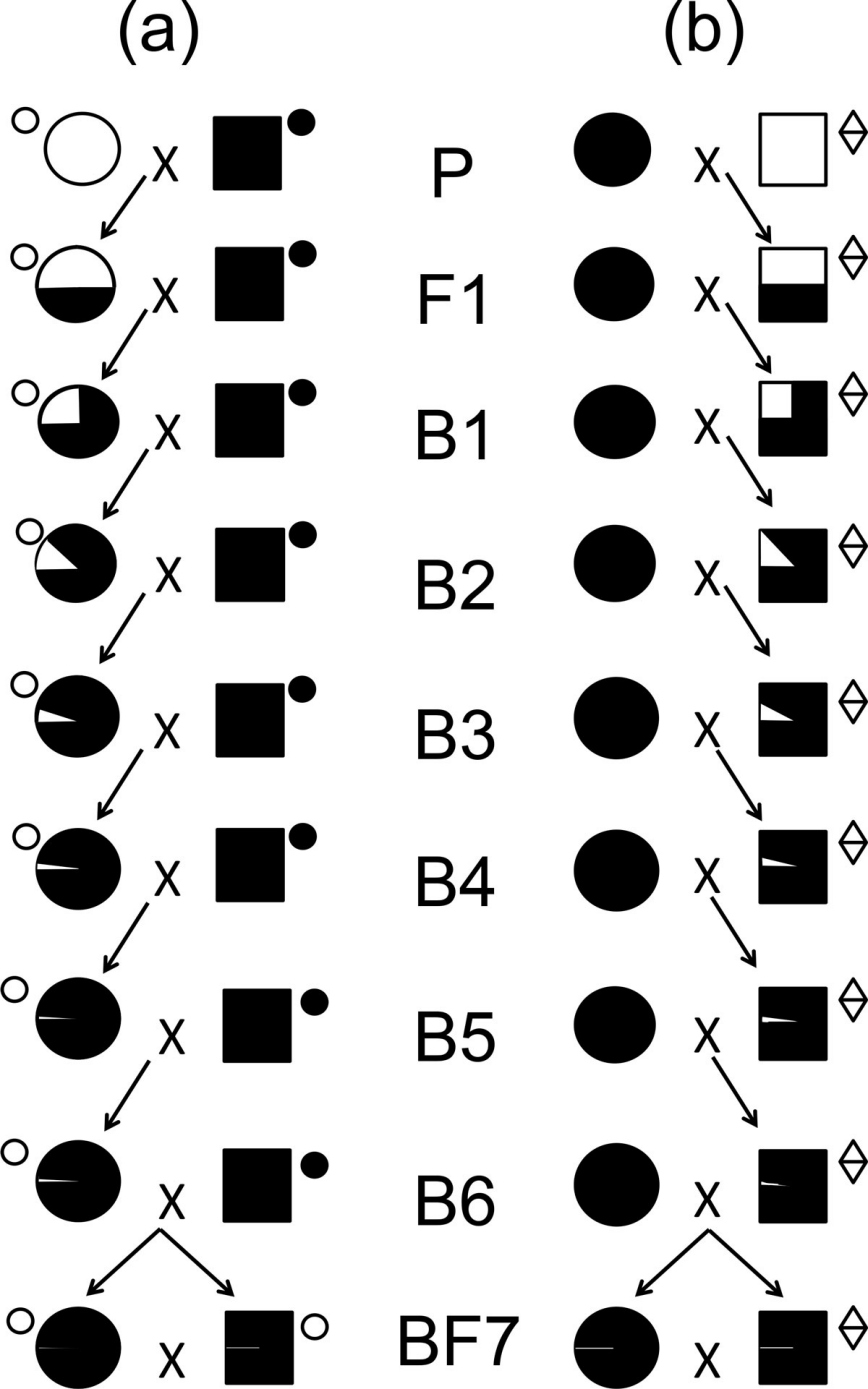
**Mating scheme for the transfer of *B. jarvisi *markers into a *B. tryoni *background**. (a) Mating scheme for transfer of *B. jarvisi *(white) mitochondria into *B. tryoni *(black) background. The mitochondrial type is indicated by small circle (white - *B. jarvisi *origin; black - *B. tryoni *origin). (b) Mating scheme for transfer of *B. jarvisi *(white) Y chromosome into *B. tryoni *(black) background. P, parents; F1, first filial generation; B1, backcross generation and number; and BF7, intercross of offspring from backcross generation 6. The proportion of the parental genomic contribution in the hybrids is indicated by the size of the corresponding black and white segments.

Crossing female *B. jarvisi *with male *B. tryoni*, then backcrossing the F1 and subsequent generations with male *B. tryoni *produces a strain of Qfly incorporating *B. jarvisi *mitochondria (Figure 4a) [32]. A simple PCR-based test is available to distinguish the mitochondria of the two species, thereby providing a protocol for distinguishing released flies from wild flies. The mitochondrially-marked *B. tryoni *stock is robust and fecund, and has been maintained in laboratory cages for 25 generations without evidence of instability. The mitochondrial marker can be incorporated into any desired Qfly strain, including the 4-way hybrids described above (Figure 2), provided that all of the females of one female parental strain in the first generation is carrying the *B. jarvisi *mitochondrion. There is a possibility that stains introgressed in this way may show some incompatibilities with the target species due to the presence of donor mitochondria, and therefore their utility requires testing under factory and field conditions.

From the reciprocal interspecific cross, female *B. tryoni *with male *B. jarvisi*, it was discovered that part of the *B. jarvisi *mitochondrial *cytb *and an intronless *tra-2 *gene were present on the *B. jarvisi *Y chromosome. This has enabled the establishment of DNA tests for the *B. jarvisi *Y chromosome [32,36]. The *B. jarvisi *Y chromosome can be incorporated into the *B. tryoni *autosomal complement by a hybridisation procedure similar to that for the mitochondrial marker, but with *B. jarvisi *used as the male parent (Figure 2b) to achieve a marked Y chromosome in *B. tryoni*. For the long-term use of such genetic markers it is, however, crucial that only sterile flies containing the markers are released so that these markers do not become established in field populations.

## Genetic sexing strains

SIT relies on the sterile males of the released strain being able to effectively compete with wild males for wild female mates [37]. In the absence of an efficient method of removing females from the release strain, mixed-sex releases must be used. Careful management of the numbers of mixed-sex release flies, to ensure overflooding of the wild population, is generally sufficient to provide control of the pest species and, in some cases, complete eradication of the invasive species has been achieved. The most outstanding example of the latter is the eradication of screwworm fly, *Cochliomyia hominivorax *(Coquerel) from the USA and most of Central America [38].

There is evidence in medfly that male-only strains are both more effective and cost efficient than mixed-sex releases. The increased efficiency of male-only releases is brought about by a number of factors: an increase in the number of matings between sterile males and wild females [39,40]; increased dispersal of sterile males in search of mates [40,41]; the elimination of potential damage to fruit caused by sterile stings of released females and consequent bacterial infection [39,42] and the reduction in costs of producing only males if females can be eliminated early in development [43,44]. To date, medfly is the only insect where GSSs have been used routinely for control of a pest species [45]. The current mass-reared SIT strains, Vienna-7 and Vienna-8, have been developed over more than 15 years. Both strains are based on Y-linked translocations involving two selectable markers - the primary marker, a fortuitously located temperture-senstive lethal (*tsl*) mutation and the white pupae (*wp*) mutation [44,46]. Considerable effort and funds from numerous international agencies have been expended on the development and maintenance of the translocation-based genetic sexing strains for medfly. Similar translocation-based strains have been developed and field-tested for *Bactrocera cucurbitae *[47,48], and *Bactrocera dorsalis *[24,49]. In *Anastrepha ludens *translocation-based GSSs have been developed and characterised but field-testing has not yet been reported [50,51].

It is of interest that in the field trials of the *Bactrocera *GSS strains, both desirable and undesirable characteristics have been identified in the SIT strains. These include low/adequate egg hatch under mass-rearing conditions [47], lower frequency of mating with wild females [52] and decreased genetic diversity but with comparable mating competitiveness of the sterile males compared to that of wild males [24]. In all of these cases the characteristics seem to be strain-specific suggesting that the selection of the strain for mass-rearing and mating competitiveness may be the most important factor to consider for either male-only or bisexual releases.

To date, the SIT program for Qfly has used mixed-sex releases. An attempt was made to develop a GSS for *B. tryoni *using translocation of visible autosomal mutations to the Y chromosome [57]. Pupal colour markers could not readily be identified because of the great variability of pupal pigmentation, which was refractory to selection. However, other markers are available, such as the chromosome 2 markers *white marks *and *bent wings *[58]. Four Y-chromosome translocations with chromosome 2 were obtained using irradiation. These were initially recovered using the chromosome 2 marker *white marks*. The translocations were then placed over the chromosome 2 marker *bent wings*, which is lethal at high temperature (31°C) at the pupal stage. Unfortunately inexplicable sterility effects involving each of the four translocations made the strains unsuitable for development as male-only release strains [57].

Fruit flies of the genus *Bactrocera *are the most important pests of horticulture in the Asia-Pacific region. The expectation is that GSSs will contribute to optimal SIT control, at the very least for reasons of cost-effectiveness and factory efficiency. However, dispersal behaviour of pests in the genus Bactrocera may be substantially different to that of medfly. For example, a number of studies indicate that newly-emerged *B. tryoni *males and females disperse further and more rapidly from a release point than does *C. capitata *(typically a kilmometre compared to about 400m; [53-56]). This means that we cannot make *a priori *assumptions about the extent of improvement from eliminating matings between sterile males and females or from increased dispersal of sterile males in the absence of concommitantly released females. It becomes critical, therefore, that more genetic-sexing strains are developed for the *Bactrocera *and are rigourously tested in field conditions. It may be that release protocols and field preformance indicators will be even more important in the *Bactrocera *than in *Ceratitis *for both mixed-sex and genetic-sexing strains.

## Isolation and application of sex-determination genes for development of genetic-sexing strains

Considerable effort is now being invested in producing male-only strains for many pest species using sophisticated molecular genetic techniques [59]. Sex-determination genes or genes with sex-specific expression have been the main targets for the design of transgene constructs which show sex-specific expression in a number of tephritid pest species such as *Anastrepha suspensa *[60], *B. dorsalis *[61] and *C. capitata *[62]. The ultimate aim is to develop transgenic sexing strains (TSS) in which the transgene acts early in development to eliminate females from the brood.

The bifunctional *doublesex *(*dsx*) gene is one of the terminal genes in the somatic sex determination pathway of the model dipteran insect, *D. melanogaster *(reviewed in [63]). The female-specific splicing of *dsx *is regulated through *transformer-2 *(*tra-2*) protein, which together with the female-specific *transformer *(*tra*) protein forms part of a multi-component spliceosome [64]. It is the non sex-specific TRA-2, which binds to the 13-nucleotide repeat elements (*dsx*RE) in the 3' untranslated region of exon 4 of *dsx *[64,65], together with the sex-specific TRA^F ^which ensures the DSX^F ^isoform is produced. The sex-specific splicing of *tra *pre-mRNA is controlled by the key gene, *Sexlethal *(*Sxl*) (reviewed in [63]). In the presence of two X chromosomes *Sxl *is set in the female-specific mode which, once established, remains in a positive autoregulatory splicing loop from stage 4 in embryonic development [66].

The primary signal of the sex determination pathway in tephritids, unlike that of *D. melanogaster*, is not the number of X chromosomes, but rather the elusive dominant male determiner, *M*, which is carried on the Y chromosome [67]. Whereas the primary signal differs, the terminal portion of the sex-determination pathway in dipteran insects other than *Drosophila *is conserved and was first shown in *B. tryoni*. Isolation of the *dsx *gene (*Btdsx*) and examination of its sex-specific transcripts also pointed to the existence of both *tra *and *tra-2 *homologues in this species [68]. The existence of *tra *in a tephritid species was first shown in *C. capitata *(*Cctra*) [69]. The pre-mRNA transcripts of *Cctra *contain TRA/TRA-2 binding sites in a region of the *tra *gene covering male-specific exons and their interspersed introns [69]. In contrast to *dsx*, the binding of TRA and TRA-2 to the *tra *pre-mRNA acts to block strong canonical splice sites, leading to the use of the weak female-specific splice sites and the removal of the strong splice sites and associated exons to yield the female-specific mRNA. Translation of the female-specific transcript encodes a longer protein product, allowing an auto-regulated supply of female-specific *tra *mRNA and TRA-F protein to be maintained in cells. Conversely, male-specific *tra *transcripts incorporate stop codons early, leading to production of a truncated and theoretically non-functional protein. Homologues of *tra *have been also been isolated from *B. oleae *[70] and *Anastrepha *species [71] and both *B. tryoni *and *B. jarvisi *[36].

**Figure 5:**
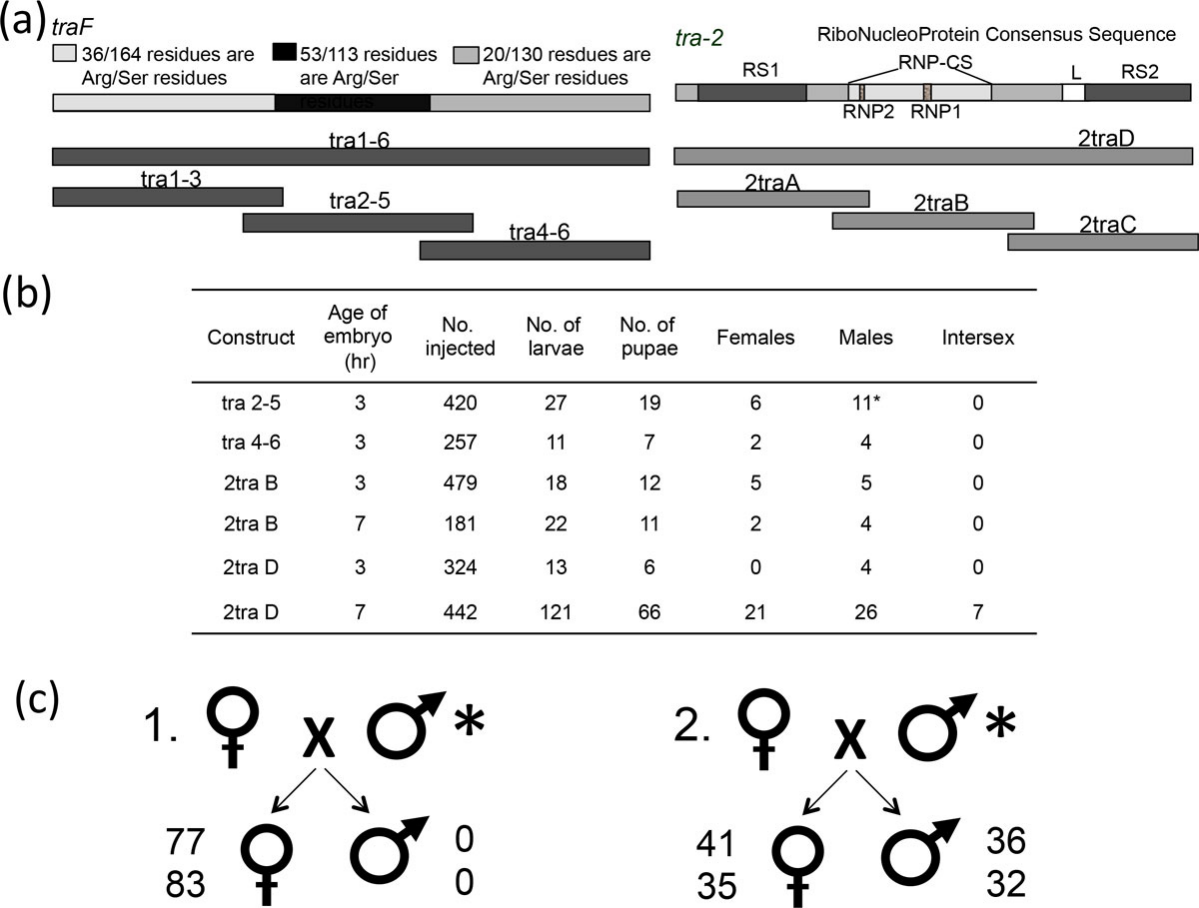
**RNAi in *B. tryoni *with *transformer *and *transformer-2***. (a) Diagramatic representation of dsRNA used to inject *B. tryoni *early embryos. Four different dsRNA fragments were designed to cover either overlapping thirds or the entire transcript of both female-specific *tra *and non-sex specific *tra-2*. (b) Preliminary results for injections of dsRNA fragments of both *tra *and *tra-2 *into either 3 hour or 7 hour embryos of *B. tryoni*. (c) Results of crosses between transformed males and wildtype females. In two cases (1) all progeny that resulted were female. In other cases (2) normal 1:1 male:female ratios were observed.

Elimination of either *tra *or *tra-2 *by RNAi early in development disrupts the mechanism favouring female-specific splicing of *tra *and *dsx *in *C. capitata, B. oleae *and *Anastrepha *spp. [69,70,72-74] and leads to the expression of *dsx*^M ^and the consequent development of male somatic tissue [75]. Preliminary assessment of *tra *and *tra-2 *in *B. tryoni *(*Bttra *and *Bttra-2*) suggests the same functionality. Injection of full-length *Bttra-2 *and partial *Bttra *dsRNA fragments into *B. tryoni *embryos of 3h and 7h AEL was performed (Figure 5), with seven intersexes recovered from *Bttra-2 *injections into 7h embryos suggesting incomplete phenotypic conversion. Complete conversion resulted from *Bttra *injections of 3h embryos, whereby two phenotypic males developed that sired all female offspring when mated to a normal female (Figure 5). Thus, either gene can independently induce sex reversal and both genes may also be useful targets for transgenic RNAi in this species. Concommitant injections of buffer showed only marginally higher pupation rates than injections with dsRNA fragments suggesting physical handling was the greatest influence on the resultant numbers of emerged adults.

Other suggested approaches have exploited tetracycline-repressible expression systems to over-express toxic or pro-apoptotic products [76,77]. An improved protocol, termed the Tet-off transgenic embryonic sexing system (TESS; [78]), tested in *A. suspensa *(Diptera: Tephritidae), utilised a genetic construct that may be applicable across a broad range of species, including *Bactrocera *species, if species-specific promoters are found and included in the transgene.

The Y-located marker in *B. jarvisi *has now been used to separate male and female embryos with both the *B. jarvisi *and the *B. tryoni *autosome set, enabling the analysis of sex-specific changes in gene expression at early stages of development [36]. Quantitative PCR pinpointed important differences in transcript abundance between the sexes over time for some sex-determination genes, including *tra *and *dsx*. These differences in transcript abundance, and the stability of *tra-2 *expression in male and female embryos over early development, suggested that *M *acts on the components of the spliceosome to simultaneously reduce both *tra^F ^*and *dsx^F ^*[36]. Combined with timing of early zygotic transcription of the important *Drosophila *sex-determination gene, *Sxl*, and the cellularisation gene *slow as molasses *(*slam*), the developmental period when *M *is active was estimated as three to six hours after egg-laying in *B. jarvisi *and from three hours up to possibly eight hours in *B. tryoni *[36].

The broad applicability of the *piggyBac*-based vector systems and the variety of cleverly designed constructs that have been shown to be functional in tephritid pest species [79] mean that the development of TSS strains for *B. tryoni*, using transgene constructs designed around the highly conserved sex-determination genes, *tra *and *tra-2*, is therefore feasible in the near future. The release of any transgenic SIT strains in Australia would, however, be subject to approval by the regulatory body.

## Genome and transcriptome analyses

The control of pest insects increasingly requires a detailed knowledge of the genome and transcriptomes; for example, the development of new genetic-sexing strains using molecular techniques requires an understanding of the sex-determination pathway, and the development of new lures requires knowledge of the olfactory system. Microsatellites have been a very important genetic tool for understanding chromosome, pedigree and population structure. The advent of next generation sequencing has opened the door to the study of the genomes and transcriptomes of non-model organisms and is becoming an increasingly important tool for understanding and manipulating pest biology and genetics, and the interactions of these with the environment.

### (i) Population structure from microsatellite sequences

Microsatellite polymorphisms were used to investigate the population structure of *B. tryoni *[80]. It was found that populations are homogeneous over large distances in north-eastern areas of Australia (Queensland), the likely ancestral home of the species. Further south (New South Wales), and to the west (Northern Territory, Western Australia), endemic populations, probably of more recent origin, were found to differ in composition from the main northern population in Queensland (Figure 1a). Smaller outbreak populations in the FFEZ and surrounding areas were found to be more closely related to the southern populations in New South Wales and to each other than to the main Queensland population [25,81,82] (Figure 1a). These results are able to inform control programs, indicating that eradicating outbreaks and their adjacent populations by exhaustive SIT would be a viable option in southern fruit-growing regions, as well as the isolated populations around Alice Springs in the Northern Territory [80].

More recently, similar findings, using microsatellite analysis, have been made for the Oriental fruit fly. *B. dorsalis *is a species with similar invasiveness to *B. tryoni*, and with similarly high population numbers and a broad distribution throughout Asia. Microsatellite analyses, albeit on smaller sample sizes, show a pattern of a large, homogeneous population in S.E. Asia with differentiated populations in more geographically isolated regions [83].

Microsatellite analyses have also been used to clarify the species status of pest fruit flies in northern Australia. *B. aquilonis *was formerly described as a separate species, with low pest status, within the *tryoni *complex, along with *B. tryoni *and *B. neohumeralis *[33]. It has been shown that *tryoni *complex flies in the north-west of Australia cannot be separated genetically from each other, regardless of minor differences in morphology, a result with important quarantine and control implications [84].

### (ii) Next Generation total genomes and transcriptomes

Early *B. tryoni *genome analysis involved cloning and sequencing of genomic regions based on similarity with *Drosophila *genes, analysis of complete and degraded *mariner *elements, and isolation of sequences containing microsatellites, reviewed in Raphael et al. [85]. A first draft, of the *B. tryoni *genome sequence has been completed and submitted to DDBJ/EMBL/GenBank (accession number JHQJ00000000, currently version JHQJ01000000). Scaffolds are based on a 80x coverage from paired-end and mate-pair Illumina sequencing, giving an N50 value of 69kb. Further, the genomes of *B. neohumeralis *and *B. jarvisi *have been sequenced and, although less complete, have been assembled *de novo *into contigs from paired-end Illumina sequencing (Gilchrist submitted). Assembled transcriptomes of various developmental stages from these three *Bactocera *species aided the creation of gene models. Continuing assembly of these genomes will be facilitated by the availablity of polytene chromosome and genetic maps for *B. tryoni *[58,86]. The genome sequences of these three related species can form the basis for resequencing comparisons of evolutionary changes, including functional modifications relating to pest status and sex determination. So far, *C. capitata *is the only other tephritid for which an assembled genome is available.

Despite the relative ease of obtaining transcriptomes, compared to complete genomes, there are relatively few published transcriptomes for tephritid species from next generation sequencing. Transcriptomes have been reported comparing wild and mass-reared medfly [87], adults and different developmental stages of *B. dorsalis *[88,89], pooled life stages of *B. oleae *[90] and sexed preblastoderm embryos of *B. jarvisi *[91]. Kumaran *et al*. [92] performed a RNA-seq analysis comparing male *B. tryoni *fed the phytochemical lure zingerone with those not exposed to zingerone. They found enrichment in gene pathways involved in energy metabolism in zingerone fed flies, as well as up-regulation of some transcripts involved in courtship and mating, which agrees with the known effects of zingerone in increasing energy metabolism and improving male competitiveness.

## Microbiomes

Microbial symbionts of tephritid fruit flies have been investigated in a number of tephritid species, and may also be exploitable for the control of pest species (reviewed in [93]). In general, symbiotic microorganisms are an important part of insect biology. Microbial symbionts, either obligate or facultative, can play a role in nutrition, reproduction, immune defense, toxin degradation and behaviour [94]. Evidence for functional roles of specific symbionts in tephritid fruit flies is scarce. Olive fly *B. oleae *is, so far, the only species in which a candidate for an obligate symbiont, "*Candidatus *Erwinia dacicola" has been identified [95,96], although this bacterium was not detected in laboratory lines of *B. oleae *[97]. It has previously been discussed that *B. tryoni *may benefit from microbial nitrogen fixation [98], while *C. capitata *appears to gain from bacteria in nitrogen fixing and pectinolase functionality [99]. However, for both species, there is no evidence for any obligate microbial symbionts. In contrast to frugivorous tephritids, closely related species within the flower head inhabiting Tephritinae subfamiliy harbor bacterial strains of "*Candidatus *Stammerula tephritidis" as co-evolved symbionts [100].

Culture dependent and independent analyses of tephritid microbiomes, including of *B. tryoni *identified variability in microbiome composition, with an overall consistent dominance of few bacterial families [101-103], primarily Enterobacteriaceae, a diverse group with members performing functions including nutrient provisioning [98,99,104].

The transmission efficiency of microbial symbionts between host individuals and generations is a key parameter defining symbiosis, and tephritids possess morphological adaptations that ensure the transmission of microbes to the next generation [105]. Many tephritid fruit flies possess an oesophageal bulb (or diverticulum), which in *B. oleae *almost exclusively harbours "*Candidatus *E. dacicola" [95,96]. Maternal transmission can be facilitated through smearing of the egg during oviposition [96,106]. Decaying fruit also plays a role in transmission of bacteria to fruit fly offspring and as a source of suitable nourishment to developing larvae [107,108].

The applied motivations for studying tephritid microbiomes so far have included the development of bacterial attractants for lure-and-kill or baiting strategies [108-113]; deprivation of beneficial bacteria that provide nutrition or breakdown insecticides [114] leading to decreased fitness in the wild; or conversely supplementation of beneficial bacteria to improve fitness of laboratory and field-release strains [115]. Poor fitness of laboratory lines, following laboratory adaptation [21] may also be an effect of microbial streamlining found in laboratory-reared insects [116], and reduced fitness subsequent to irradiation treatment [117,118] may derive in part from distortion of the microbial diversity [119].

A facultative symbiont worth noting is the maternally inherited intracellular *Wolbachia *(Alphaproteobacteria). *Wolbachia *is well known for manipulating host reproduction, commonly by cytoplasmic incompatibility (CI) [120]. In CI, the outcome is crossing sterility between infected males with females that are either uninfected or infected with a different, incompatible strain of *Wolbachia*. A male-only *Wolbachia *infected fly line may be released for population control, and this was first field tested as incompatible insect technique (IIT) for naturally infected *Rhagoletis cerasi *(reviewed in [121]), as an alternative to radiation-based SIT. IIT has been further developed, e.g. through the successful transfer of non-native *Wolbachia *strains into uninfected medfly and olive fly, and resulted in population suppression in cage experiments [122-124]. IIT requires male-only releases or an effective way of sterilising released females in order to avoid the establishment of the released *Wolbachia *strain in the field. A way to overcome this issue could be the combination of IIT with SIT, also with a more favourable lower radiation dose that may have reduced fitness costs [5,125]. Release of *Wolbachia*-infected low dose irradiated males would provide CI with field females while released infected females would be impaired in fertility due to low dose radiation. As an additional advantage, released flies carry detectable *Wolbachia *without the need to be further marked.

Research towards applications in *B. tryoni *has shown that this species harbours *Wolbachia *infections at very low prevalence in tropical northern Queensland while populations from temperate regions are uninfected [126]. Strikingly, within the tropics, *B. tryoni *shares identical *Wolbachia *with five *Bactrocera *species (including *B. neohumeralis*) and *Dacus axanus *[126]. This tropical restriction of *Wolbachia *in Australian tephritid fruit flies is unusual and warrants further investigation, particularly as the role of *Wolbachia *in Australian tephritids is still unclear. It is likely that frequent ecological interactions of tropical Australian fruit flies (e.g. in shared host fruits or through parasitoid wasps) results in frequent exposure of tephritid species to *Wolbachia *resulting in horizontal *Wolbachia *transmission [126].

## Conclusions

A range of genetic tools is now available for *B. tryoni *that will inform the development of improved SIT strains. This is particularly relevant with the recent announcement of investment in a new SIT facility and research to develop a male-only strain. Performing 4-way crosses would be a relatively straightforward way, requiring few resources, to develop a more robust mass-rearing strain, overcoming problems of inbreeding, and can be applied to any species. Genetic markers for identification of sterile from wild flies can be introduced into mass-reared strains via the 4-way hybrid method. These markers can include the mitochondrion or Y chromosome from a related species, *Wolbachia *or genetic material introduced by germ-line transformation. Some of the genes of the sex-determination pathway have now been isolated and expression data obtained which, together with the availability of germ-line transformation, opens the way for genetic manipulation to produce male-only broods. Ultimately, genetic modification for male-only strains could be introduced into optimal 4-way hybrid strains. Release of the first draft of the genome assembly and imminent publication of further transcriptomes means that important gene families will now be characterised such as genes controlling odorant perception and processing, sex-determination and immune response. The ability to combine expression information from transcriptomes and genome sequence information will enable the identification of gene promoter regions e.g. embryo-specific enhancers for the manipulation of expression of sex-determination genes are an important tool for the generation of TSS. Importantly the genomes of related pest species are also available, albeit at lower coverage at this stage, so that comparisons and genetic experiments between the species will identify the genetic bases of differences in pest status, mating behavior, odorant perception etc. Much still needs to be learned about how the microbiome in *B. tryoni *is shaped and how it affects fly fitness. High throughput characterisation of the microbiome of flies from different environments in combination with fitness studies will lead to the identification of beneficial microbial isolates that may be used to improve rearing and release programs. Future studies will need to further investigate the unusual distribution of *Wolbachia *in Australian tephritids, and laboratory experiments are needed to test the role and applicability of this promising bacterium in manipulating reproduction and fitness.

## List of abbreviations used

FFEZ: Fruit Fly Exclusion Zone; GSS: Genetic sexing strain; ITT: Incompatible insect technique; PCR: Polymerase Chain Reaction; Qfly: Queensland fruit fly; SIT: Sterile Insect Technique; TESS: Tet-off transgenic embryonic sexing system; TSS: Transgenic sexing strains.

## Competing interests

All authors declare that they have no competing interests
